# Parasite Population Genetic Contributions to the Schistosomiasis Consortium for Operational Research and Evaluation within Sub-Saharan Africa

**DOI:** 10.4269/ajtmh.19-0827

**Published:** 2020-05-12

**Authors:** Joanne P. Webster, Maria Inês Neves, Bonnie L. Webster, Tom Pennance, Muriel Rabone, Anouk N. Gouvras, Fiona Allan, Martin Walker, David Rollinson

**Affiliations:** 1 Department of Pathobiology and Population Sciences, Centre for Emerging, Endemic and Exotic Diseases (CEEED), Royal Veterinary College, University of London, Hawkshead Campus, Herts, United Kingdom;; 2 London Centre for Neglected Tropical Disease Research (LCNTDR), Imperial College Faculty of Medicine, London, United Kingdom;; 3 Department of Life Sciences, Wolfson Wellcome Biomedical Laboratories, The Natural History Museum, London, United Kingdom;; 4 School of Biosciences, Cardiff University, Cardiff, United Kingdom

## Abstract

Analyses of the population genetic structure of schistosomes under the “Schistosomiasis Consortium for Operational Research and Evaluation” (SCORE) contrasting treatment pressure scenarios in Tanzania, Niger, and Zanzibar were performed to provide supplementary critical information with which to evaluate the impact of these large-scale control activities and guide how activities could be adjusted. We predicted that population genetic analyses would reveal information on a range of important parameters including, but not exclusive to, recruitment and transmission of genotypes, occurrence of hybridization events, differences in reproductive mode, and degrees of inbreeding, and hence, the evolutionary potential, and responses of parasite populations under contrasting treatment pressures. Key findings revealed that naturally high levels of gene flow and mixing of the parasite populations between neighboring sites were likely to dilute any effects imposed by the SCORE treatment arms. Furthermore, significant inherent differences in parasite fecundity were observed, independent of current treatment arm, but potentially of major impact in terms of maintaining high levels of ongoing transmission in persistent “biological hotspot” sites. Within Niger, naturally occurring *Schistosoma haematobium/Schistosoma bovis* viable hybrids were found to be abundant, often occurring in significantly higher proportions than that of single-species *S. haematobium* infections. By examining parasite population genetic structures across hosts, treatment regimens, and the spatial landscape, our results to date illustrate key transmission processes over and above that which could be achieved through standard parasitological monitoring of prevalence and intensity alone, as well as adding to our understanding of *Schistosoma* spp. life history strategies in general.

## INTRODUCTION

Schistosomiasis is a neglected tropical disease, caused by dioecious blood flukes of the genus *Schistosoma*, estimated to currently infect more than 220 million people.^1^ The disease burden is greatest (at least 90%) within sub-Saharan Africa (SSA), where the main species causing human schistosomiasis are *Schistosoma mansoni* (a causative agent of intestinal schistosomiasis) and *Schistosoma haematobium* (and hybrids therein, the causative agents of urogenital schistosomiasis), transmitted via eggs excreted in feces or urine, respectively. After 60 years of major multidisciplinary control efforts, great success has been achieved against *Schistosoma japonicum* (a causative agent of intestinal schistosomiasis) within China, and actual elimination/interruption of transmission has been achieved in Japan. However, although efforts to control *S. mansoni* and *S. haematobium* through large-scale preventive chemotherapy (PC)/mass drug administration (MDA) with praziquantel (PZQ) across SSA have also had a substantial impact on preventing or relieving morbidity,^2^ considerable work is still needed to achieve elimination.

Contributing to this challenge, a recent work serves to highlight that schistosomes are highly complex organisms, and numerous essential characteristics of their biology and epidemiology remain unknown. Many of these unknowns potentially pose major challenges for sustainable disease control and certainly for the interruption of transmission. For instance, with more sensitive diagnostic surveillance tools, there are reports that prevalence levels are substantially greater than previously thought,^3^ especially among preschool-aged children and infants.^4^ There is also evidence of maintenance and reemergence of schistosomiasis in previously controlled regions, exacerbated, at least in part, through wildlife and/or domestic animal reservoirs, not only in Asia^5–7^ but also it is becoming increasingly apparent within Africa too.^8,9^ Linked to this, it is also now known that hybrid/introgressed *Schistosoma* species are common across several high-endemicity regions of SSA,^10^ especially West Africa,^11–13^ and have recently spread into Europe.^14^ Perhaps, most disconcerting of all in terms of achieving the World Health Organization (WHO) goals of sustainable control, elimination of schistosomiasis as a public health problem, and ultimately interruption of transmission is the potential of reduced drug efficacy among populations under high chemotherapeutic pressure.^15^ Yet accurate interpretation of prevalence and intensity data is often hampered because of the poor sensitivity of traditional parasitological diagnostics, particularly at low intensities of infection.^16^ Finally, there remain many critical unresolved questions on the schistosomes’ resilience to the various interventions imposed on them.

**Figure 1.**
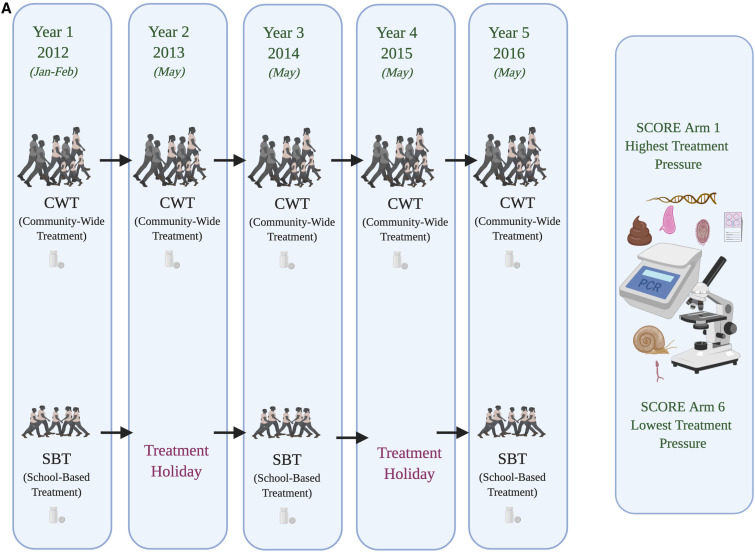

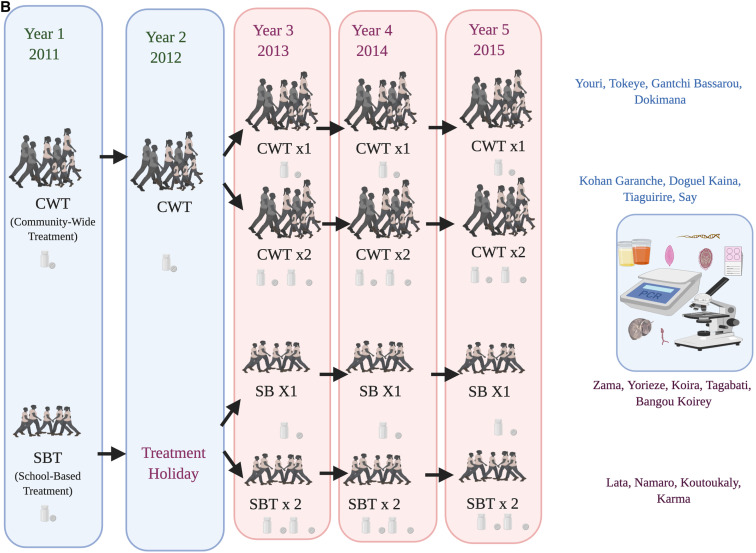

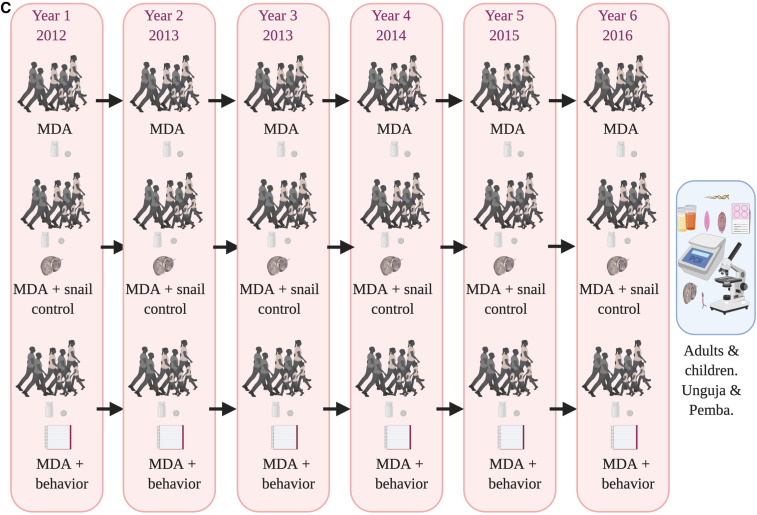
Schistosomiasis Consortium for Operational Research and Evaluation treatment program designs incorporating population genetic analyses encompassing: (**A**) school-based vs. community-based mass drug administration (MDA) *Schistosoma mansoni* control in Tanzania, (**B**) biannual vs. annual MDA across school-based and community-based *Schistosoma haematobium* control in Niger, and (**C**) biannual MDA alone vs. biannual MDA + snail control vs. biannual MDA + behavioral change elimination interventions on *S. haematobium* in Zanzibar.

The “Schistosomiasis Consortium for Operational Research and Evaluation” (SCORE; https://score.uga.edu/) was designed to conduct large-scale operational health research into morbidity control and elimination.^17–19^ The overall aim within the SCORE gaining and sustaining control studies was to evaluate different schedules for providing PC through MDA either delivered through community-wide treatment (CWT) or through school-based treatment (SBT) (Figure 1A and B). Analyses of the population genetic structure of schistosomes under these contrasting scenarios were proposed to contribute supplementary critical information with which to evaluate the impact of large-scale control activities and ideally guide how activities could be adjusted. In particular, it was predicted that population genetic analyses should reveal novel information on effective parasite population size and any bottlenecks imposed, the role of refugia, recruitment and/or ongoing transmission of genotypes, occurrence of hybridization events, differences in reproductive mode, and degrees of inbreeding, as well as fitness traits of individual parasites that may be evidenced through sibship analyses, impact of drug holidays (predicting differential bounce back under different times), and, hence, overall, the evolutionary potential of parasite populations under contrasting drug pressures. Furthermore, it was predicted that applying population genetic analyses to such parasite populations under contrasting treatment pressures should add to our understanding of their life history strategies and to the importance of ecological and evolutionary theory in general.

The initial SCORE population genetic subcomponents focused on villages in the gaining control studies where baseline infection prevalence levels were at least 25%, and specifically those two of the six intervention arms with the most contrasting schedules of PZQ pressure: CWT every year versus SBT every other year. This design was followed throughout for *S. mansoni* in Tanzania, with samples collected from school-aged children (age [predominantly] 9–12 years) before MDA from areas with intestinal schistosomiasis (but low or zero co-endemic urogenital schistosomiasis) close to Lake Victoria (Figure 1A). In Niger, initial sampling focused on school-aged children (age 9–12 years) from the Kolla and Say districts of the Niger River valley, regions of high urogenital endemicity (but little or zero co-endemic intestinal schistosomiasis). However, midway through the study, because of issues of randomization within the main Niger SCORE program, the study design was changed to evaluate biannual treatment to annual treatment (CWT × 2 versus CWT × 1, SBT × 2 versus SBT × 1; Figure 1B). In addition, from 2011, population genetics studies were also included as a component of the SCORE elimination study within the “Zanzibar Elimination of Schistosomiasis Transmission” (ZEST) alliance. Zanzibar is composed of an archipelago including the two main islands, Unguja and Pemba, located approximately 35–60 km off mainland Tanzania. The relative isolation of these island populations makes them particularly suitable for assessing disease control strategies, complemented by an apparent lack of potential animal host reservoirs/zoonotic schistosomiasis transmission (but see ref. 20) and an *S. haematobium* population genetic lineage distinct from that of much of mainland Africa.^21^ The overreaching aim of ZEST was to evaluate the ability of contrasting intervention strategies to achieve elimination as a public health problem on Pemba and complete interruption of transmission on Unguja within 5 years.^18,22–24^ The cluster randomized trial involved three study arms, implemented across 45 randomly selected shehias (small administrative regions) on each island, to assess the differential impact of the following: 1) biannual MDA alone, 2) biannual MDA + snail control, and 3) biannual MDA + behavioral interventions (Figure 1C).

Here, we summarize and discuss some of the key findings to date from these studies and how they contribute to our understanding of the population genetics of schistosomes in response to differential control selection pressures. We also identify some of the main continuing challenges and discuss how they relate to the interpretation of standard monitoring and evaluation data, as well as the feasibility of achieving the disease control targets.

### The need for new tools to answer more complex questions.

The two most commonly used indicators to determine the impact of schistosome control programs are prevalence and intensity of infection (typically measured as eggs per gram [EPG] of feces for intestinal schistosomes and eggs per 10 mL of urine for urogenital infections). Intensities of infection are assumed to relate indirectly to the degree of host morbidity and directly to the hosts’ potential contributions to transmission. Prevalence and intensity data can also be used to evaluate the efficacy of treatment through the cure rate and the egg reduction rate (ERR), the latter recommended by the WHO.^25^ Cure rate is a measure of the percentage of individuals that no longer excrete eggs after treatment compared with baseline, whereas the ERR measures the percentage reduction in the number of eggs passed.^26^ Both of these measurements are subject to the sensitivity and specificity of the assay used. Reduced or suboptimal drug efficacy in a population is flagged when the observed ERR is less than the reference/expected value by at least 10%.^25^ Only recently have statistical methods been implemented to estimate and understand variability in drug efficacy among individuals, including identification of individual and trial design factors associated with treatment response.^27^ Efforts are underway to collate, standardize, and analyze, on a global scale, individual data on responses to antischistosomal treatment, with goals that include the optimization of treatment regimens, the development of enhanced efficacy monitoring strategies, and, ultimately, new approaches to mitigate the risk of emerging drug resistance.^28^ However, even with improved statistical tools, and the burgeoning use of mathematical transmission models to predict the impact of intervention strategies and inform appropriate, achievable control, and elimination goals,^29,30^ empirical information on the impact of MDA on parasite populations remains fundamentally limited by the reliance on these indirect measures of infection, that is, egg counts. For schistosomes, this is further exacerbated as direct quantification, and/or molecular characterization, of adult worm burden from within humans is not feasible because of the inaccessibility of adult worms within their intravascular locations (unlike that for many of the soil-transmitted intestinal helminths, which can be obtained by chemoexpulsion). This raises fundamental and unresolved questions on the population biology of schistosomes that could play a key role in shaping the transmission dynamics of the parasite and its potential response or resilience to the treatment.^31,32^

In the past, population genetic analyses of schistosomes relied on the infection of laboratory-reared snails to obtain cercariae, infecting laboratory-reared rodents and then obtaining the passaged adult worms by perfusions. However, with the development of novel methodologies for sample storage of single miracidia and cercariae stages collected directly from natural human and snail infections, respectively, on Whatman FTA cards (GE Healthcare Life Sciences, Buckinghamshire, United Kingdom), these logistical, ethical, and biological caveats have been circumvented.^33,34^ Additional refinements developed through SCORE studies and beyond now allow repeat analyses on unamplified genomic DNA, cost-effectively and without genome amplification bias, thereby improving data quality and giving the potential for greater analytical depth.^35,36^ Such new methodologies and refinements allow an enormous reduction in the logistical effort required in assaying parasite populations. Furthermore, these methodologies can be applied to natural schistosome populations across continents, allowing wide-scale genetic and genomic analyses of schistosome populations over space and time, and encompasses all life stages from adult worms to free-living miracidia and cercariae (e.g., refs. 37–39).

Microsatellites remain one of the most powerful Mendelian markers currently available, although the mutation rate varies across loci, and are ideal for population genetic studies to identify clusters of genetically related individuals. This is potentially indicative of population-level differences over space and time, as well as bottleneck effects, and of both historical and contemporary population-level changes. A number of *Schistosoma* species–specific microsatellite markers have thus been developed and evaluated—from those for *S. japonicum* in Asia^5,6^ to *S. mansoni*^40–42^ and, more recently, *S. haematobium.*^40,43,44^ For the SCORE Tanzanian *S. mansoni* samples, two multiplex microsatellite assays were developed from 17 previously published microsatellite loci, which spanned the *S. mansoni* genome.^40–42^ Following data cleaning and quality assessment, 13 of the 17 microsatellites loci were successfully amplified. Similarly, for the SCORE samples from Niger and Zanzibar, we developed two novel multiplex microsatellite assays to enable cost-effective simultaneous amplification of 18 informative microsatellite loci of *S. haematobium* populations.^39,45^ However, the cross-reactivity of these *S. haematobium* microsatellite loci with other medically and veterinary important schistosomes across the closely related *S. haematobium* group species required additional consideration, particularly given the ability of species within this group to hybridize.^12^ The advances in the DNA preparation of schistosome larval stages stored on Whatman FTA cards further enabled both the replication within technique and also the use of additional tools and techniques to be performed on the same individual *Schistosoma* larval samples. In particular, we were thus able to include species diagnostic PCR’s and DNA sequencing through analysis of mitochondrial *cox*1 and nuclear ITS regions on the Nigerian samples.^11^

### Key findings to date from the SCORE population genetics studies.

Key results to date of the SCORE population genetic datasets indicate that, within the Tanzanian studies, there was little or no differential impact of annual CWT or SBT on the population genetic structure of *S. mansoni*, with no significant clustering or genetic differentiation by treatment or by year. Instead, it appeared that a large refugia of parasites from untreated individuals in the community and/or in neighboring communities undergoing different treatment schedules allowed for high levels of gene flow and, hence, a mixing of the parasite population between years and between sites. Any observed genetic differentiation at the village or school level appeared most likely to be explicable by the eco-epidemiological location itself (such as distance to water or human population movement), rather than the SCORE treatment arm. This is consistent with the previous work that observed that most of the genetic diversity in *S. mansoni* occurs at the human host level, rather than at the village or district level.^44,46,47^ Infrapopulation genetic diversity is usually interpreted as the combined outcome of the genetic diversity of parasites circulating in the environment, differences in exposure due to variability in host behaviors, particularly those related to water contact; that is, location, duration, and time of day, as well as parasite establishment, often dependent on host gender and immune response. These factors could well be predicted to dilute any effect imposed by the SCORE-contrasting treatment arms, particularly under conditions of the high gene flow that was apparent across these lacustrine Tanzanian populations.

Within the SCORE Niger data sets, there were also no significant differences observed between the original SBT and CWT in terms of genetic diversity and/or bottlenecking effects. However, although there appeared to be no significant clustering or genetic differentiation by CWT × 1 and CWT × 2 treatment arms, the greatest reduction in overall genetic diversity levels was observed by the end of the study between the SBT × 2 relative to SBT × 1, potentially consistent with the greater drug pressures (SBT × 2) imposed on these groups. Furthermore, an extensive proportion of hybrid schistosomes, rather than *S. haematobium*, within many of the Niger sites was apparent.

In the SCORE Zanzibar elimination study within ZEST, the greatest differences overall, both in terms of genetic sub-structuring and parasite fecundity (see in the following text), were observed between the islands, consistent with Pemba’s higher mean infection intensities than Unguja.^18^ Population genetic analyses also revealed a number of important issues that could not have been detected by classical parasitological prevalence and intensity measures alone. For instance, the key outcome of the main SCORE study was that, although MDA substantially reduced the *S. haematobium* prevalence and infection intensity, and, hence, “elimination as a public health problem,” it was insufficient to interrupt transmission either alone or when combined with either snail control or behavioral change activities.^18,22^ Yet, ongoing population genetic analyses indicate the strongest selective pressures placed on these *S. haematobium* populations, as suggested by both genetic diversity and outbreeding estimates, together with reductions in the estimated number of adult worms present within each child/adult host, were observed in the MDA + snail control arms. Furthermore, on each island, it appeared that many of the shehias with the highest fecundity levels went on to become persistent hotspots (PHS) relative to those where control was successful. These findings thus highlight the additional contributions that population genetic analyses can make in elucidating biological drivers, and potential gene–environmental interactions, that can lead to areas remaining PHS^48,49^ despite ongoing control measures.

However, even with such sensitive and specific population genetic tools, interrogation of these SCORE datasets also highlighted that many challenges remain for analyses and interpretation. For instance, one of the original aims of SCORE population genetic analyses was to examine the differential impact of drug pressure on the potential for bottlenecking events as well as effective population sizes (*Ne*) (or the effective number of breeders contributing to the next generation [*Nb*]) of the remaining schistosomes. Analyses of both Ne/Nb within both our SCORE *S. haematobium* datasets from Zanzibar and Niger (unpublished observations) using two available distinct software packages produced notably different patterns, resulting in too much uncertainty to allow conclusions to be drawn. It was evident that further tools and analytical development would be required for the *Schistosoma* system to enable robust estimates of *Ne/Nb*. Indeed, a previous study that used parentage analysis to quantify family structure (including *Nb* and the number of full-sib families) showed that the reproductive success of breeding schistosomes was likely skewed, resulting in differential representation of each family in the offspring pool.^32^ It is thus likely that different reproductive outputs could affect the results of statistical models used to estimate female worm burdens from miracidial offspring data using parentage analysis.^31^ Similarly, sibship reconstruction is a form of parentage analysis that can be used to identify the number of helminth parental genotypes infecting individual hosts using genetic data on only their offspring. However, methods for inferring worm burdens from sibship reconstruction data on numbers of unique parental genotypes were lacking, limiting the method’s scope of application. Therefore, to proceed with our SCORE population genetic analyses, further statistical methods for estimating female worm burdens from data on the unique number of distinct female parental genotypes derived from sibship reconstruction were developed^31^ and applied to both the *S. haematobium* and *S. mansoni* SCORE datasets to produce more robust measures on the within-host parasite populations present.

### The challenge of density dependence and differential fecundity.

**Figure 2.**
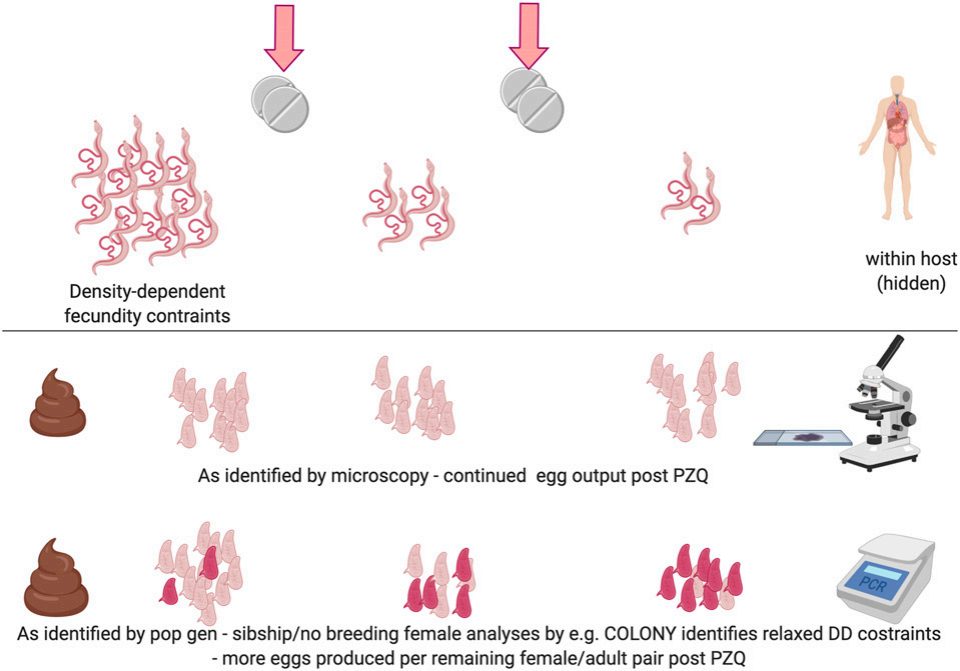
Identification of density-dependent fecundity changes after treatment with praziquantel (PZQ) as revealed by population genetic analyses but not by standard (monitoring and evaluation) parasitological microscopy. In high worm-burden host environments, density-dependent fecundity down regulates the per capita egg contribution of each male–female worm pair. Treatment with PZQ reduces the size of the parasite population, relaxing density-dependent inhibition of parasite fecundity. This enables increased egg production in drug-resistant or reduced-susceptibility parasites that survive treatment. Consequently, microscopy identifies only a limited reduction in egg output, whereas population genetic analyses allow direct identification of the number of female worms. The combined use of microscopy and population genetic techniques permits estimation of the egg output per female worm.

Potential density dependence among schistosome populations raises a number of important public health implications. Density dependencies can regulate parasite reproduction, govern their transmission dynamics, influence the interpretation of standard parasitological egg count data collected during monitoring and evaluation activities (because using egg counts as a proxy for worm burden depends on the relationship between these two stages in the parasite lifecycle), and can enhance resilience to interventions. Density dependence can be either positive (density-dependent facilitation) or negative (density-dependent inhibition). For example, in dioecious organisms such as schistosomes, the probability of a female worm encountering a male worm and forming a mating pair may be so low at low parasite densities that reproduction is restricted, whereas at higher parasite densities, the probability of mating pair forming and successful reproduction occurring increases—hence positive density-dependent facilitation. By contrast, in negative density-dependent inhibition, parasite population growth is inhibited at higher parasite densities because of a reduction in per capita egg contribution by each female worm (= adult worm pair) as a function of within-host infection intensity (Figure 2). Competition due to limited host resources such as space and nutrients, as well as immunological host responses, are possible mechanisms driving this process.^50,51^

Whether and to what extent density-dependent processes regulate the fecundity of schistosomes is an unresolved, longstanding question because, as stated earlier, it is not possible to directly quantify the relationship between *Schistosoma* egg counts and worm burdens (at least within human hosts). Furthermore, there appears to be no simple worm–egg/larva relationship because of either the trapping of eggs in host tissues and/or to the occurrence of density-dependent mechanisms. The strongest direct evidence for density-dependent fecundity inhibition in schistosomes was generated over 40 years ago, from two human autopsy studies providing data on *S. mansoni* and *S. haematobium* worm burdens and associated egg counts.^52,53^ However, further statistical analyses of these datasets have provided controversial and contradictory conclusions.^54–56^ More recent studies have taken a different approach, by relating *S. mansoni* in egg counts to circulating anodic (CAA) and cathodic (CCA) antigens, glycoconjugates secreted from the gut of schistosomes, and found the slopes less than unity, suggestive of density dependence.^57,58^ Still, the existence of a direct relationship between CAA or CCA antigens and worm burdens has not been proven in humans because it is not possible to exclude other mechanisms affecting antigen production and clearance.^58–60^

One recent population genetic study, however, estimated the number of adult worm genotypes of *S. mansoni* in Tanzanian children using parentage analysis and sibship reconstruction from microsatellite analyses of miracidia.^47^ Analyses using our newly developed statistical tools indicated that, despite the (minimum) number of adult worm pairs present in school-aged children having reduced over time in response to PZQ treatment, there was little or no evidence of any corresponding reduction in parasite infection intensities, as measured by EPG. This indicated there was a relaxation of the density-dependent inhibition on schistosome fecundity following treatment with PZQ among these *S. mansoni* populations.^31^ Furthermore, it highlighted how the continued high EPG infection intensity levels observed after PZQ treatment could have, without the added value of population genetic analyses, been erroneously otherwise interpreted as evidence of, for instance, either lack of treatment coverage and/or reduced drug efficacies if measured only through standard monitoring and evaluation parasitological protocols. Nevertheless, it must also be emphasized that where interventions, through reductions in size of the parasite population, relax density-dependent inhibition in parasite fecundity, as observed here, this can also subsequently lead to increased reproduction rates in drug-resistant or reduced-susceptibility parasites that survive treatment.^61^

Our ongoing analyses of SCORE *S. haematobium* populations from Zanzibar appear, however, to indicate that such density dependence in response to PZQ/control pressures may not be universal because there was no obvious evidence of relaxed density-dependent inhibition in parasite fecundity in relation to intervention arm. Rather, it appeared that both the number of estimated adult worms and the estimated egg output per female worm significantly reduced across all arms on both islands. Although this could potentially reflect species differences between *S. haematobium* and *S. mansoni*, consistent with their contrasting mean daily egg output per adult worm, it is important to acknowledge that the aforementioned Tanzanian *S. mansoni* populations represented a true pretreatment baseline population,^44^ whereas these Zanzibar *S. haematobium* populations had been subjected to many previous treatments, over several years, with PZQ before the start of the SCORE/ZEST program.^62^ Indeed, within these Zanzibarian *S. haematobium* populations, there was evidence of density dependence within individuals in relation to host age and gender (and, hence, also infection intensity profiles), consistent with that previously documented through population genetic analyses for both *S. mansoni* in Tanzania^31,47^ and *S. haematobium* in Mali.^44^ Furthermore, population genetic and statistical analyses did indicate inherent differences in schistosome fecundity levels by site across both islands, frequently consistent with those shehias that went on to be classed as nonresponder hotspot sites. Thus, although where and under what conditions density dependence regulates schistosome transmission dynamics remains complex, and certainly further work is required in terms of its measurement (including, despite its additional logistical challenges, in terms of maximizing miracidial sampling over multiple time points per host), we have demonstrated its existence and potential for the interpretation of, and resilience within, public health interventions. Likewise, our results to date do emphasize that potential variability in density-dependent process must now be incorporated into those mathematical transmission models which are being increasingly used to inform intervention policy and design decisions.

### The challenge of interspecific hybridization.

**Figure 3.**
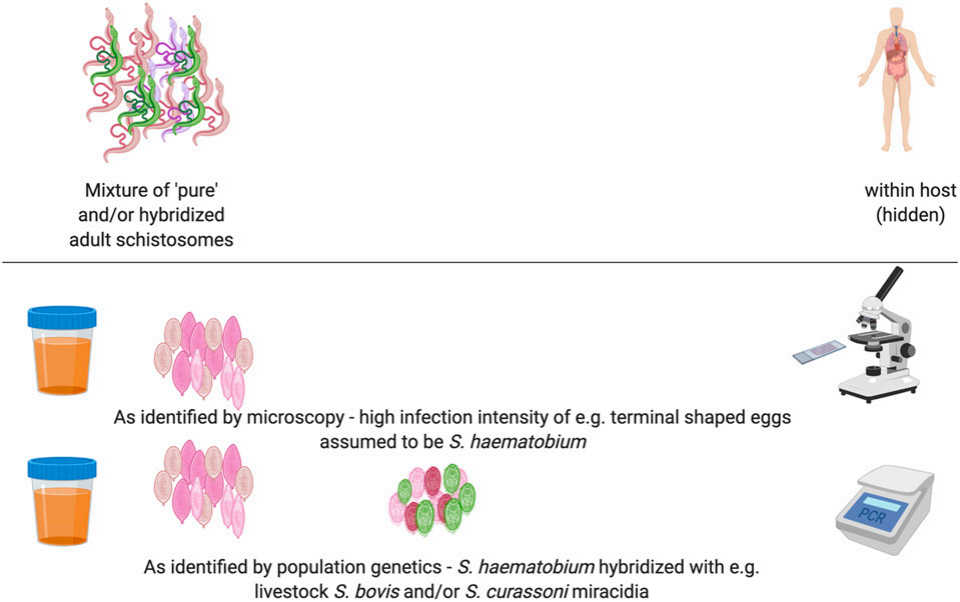
Identification of infecting species/hybridized species combinations as revealed by population genetic analyses but not by standard (monitoring and evaluation) parasitological microscopy. Hybridization and introgression can occur when closely related schistosome species coinfect a host. In humans coinfected with different species or hybridized schistosomes, standard parasitological microscopy only provides information on egg output and egg shape. Population genetic analyses in hatched miracidia can identify, for example, here *Schistosoma haematobium* hybridized with livestock *Schistosoma bovis* and/or *Schistosoma curassoni*.

One of the most notable results to date with the SCORE population genetic analyses from Niger was the consistently high proportions, often around 80%, of hybridized *Schistosoma* combinations observed, specifically that of *S. haematobium* with *Schistosoma bovis* hybrids, in comparison with the pure *S. haematobium* originally targeted for investigation. Furthermore, most of the infected *Bulinus* snails across all sites were found to be transmitting the livestock schistosome *S. bovis* alone, with the remainder, from only five villages north of Niamey, shedding hybrids and only very few shedding *S. haematobium.* Hybridization of parasites is an emerging public health concern at the interface of infectious disease biology and evolution. Increasing economic development, human migration, global trade, and climate change are all modifying the geographic distribution of existing human, livestock, companion animal, and wildlife parasites.^10^ As a result, human populations encounter new infections more frequently, and coinfection by multiple parasites from different lineages or species within individual hosts can occur. When closely related species, such as within the *S. haematobium* group here, coinfect a host, introgression can result (the introduction of single genes or chromosomal regions from one species into that of another through repeated backcrossing), and whole genome admixture can occur through hybridization. Such hybridization can have major implications in light of the current global push for human disease control programs to shift from controlling morbidity to interrupting transmission.^2,9^ To what proportion, and where, such hybrids are ancient or ongoing,^2,9,63–65^ how such introgression may alter host range or host morbidity, and what this implies regarding our concept of species within the *Schistosoma* system and beyond are critical areas to explore in future research. The challenge of hybridization of schistosomes for routine MDA monitoring and evaluation of parasitological-based diagnostics, however, versus molecular typing diagnostics, is illustrated in Figure 3.

### The challenge of PZQ resistance.

Last, but by no means least, is the role population genetic (and genomic) analyses can play in elucidating the sustainability of any drug essential for PC/MDA. Drug efficacy, or drug resistance, could not be directly examined within the overall SCORE framework because of the inability to interfere with the timings of the treatment schedule and ethical requirements regarding the need to treat those found to be infected. Nevertheless, the potential importance of drug resistance for operational research and effective disease control, and the need to predict and/or detect as it emerges, cannot be overstated. Drug resistance has been the bane of veterinary anthelmintic treatments, and for human schistosomiasis, this is a particularly pertinent issue, given that PZQ is currently the only drug available for PC. Although the genetics of anthelminthic resistance in general are poorly understood, this is particularly true in regard to the genetics of PZQ resistance in schistosomes. Indeed, even the potential mechanism of action of PZQ remains uncertain.

**Figure 4.**
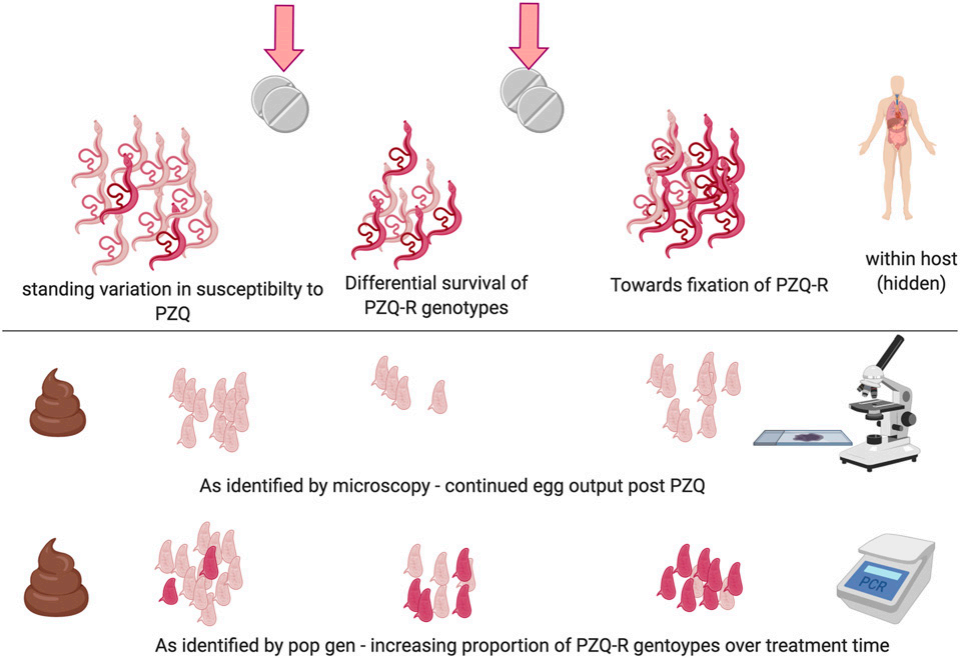
Identification of potential praziquantel (PZQ)-resistant parasites as revealed by population genetic analyses but not by standard (monitoring and evaluation) parasitological microscopy. Parasites with reduced susceptibility to PZQ are more likely to survive exposure to the drug, increasing the frequency of reduced-susceptibility/resistant variants (PZQ-R) in subsequent generations. Standard microscopy identifies sustained egg output after treatment, which may be indistinguishable from relaxation of density-dependent fecundity. Population genetic analyses can identify changing genetic structure and ultimately quantify the frequency of PZQ-R genotypes.

Resistance generally arises through repeated and extensive exposure of the parasite to the selective pressure of a chemotherapeutic agent. Parasites that survive multiple exposures to a given drug may pass on genetic variants that make the offspring more resistant to the drug. The rate and likelihood of resistance developing is influenced by a broad range of factors including, but not exclusive to, baseline parasite genetic structure and underlying standing variation in drug tolerance on which selection can act, effective population size, refugia, and gene flow in parallel to treatment frequency, dosage, and whether single or combination drug regimens.^66,67^ Figure 4 demonstrates how population genetics can, nevertheless, help evaluate and contribute to the detection of potential changes in drug resistance efficacy.

Given this inherent range of factors influencing the rate and likelihood of PZQ resistance emerging, as may be predicted, population genetic data gathered to date can be contradictory, particularly in relation to the time of assessment. For example, *S. mansoni* collected 4–6 weeks after treatment in Brazil has been reported to show reduced genetic diversity,^68^ with a similar reduction in *S. mansoni* genetic diversity 6 months after a single PZQ treatment in two schools in Tanzania.^69,70^ However, genetic diversity of *S. mansoni* from these same regions of Tanzania over a 5-year period of MDA was not found to be reduced.^47^ Similarly, one longitudinal study examining the genetic diversity and population structure of *S. mansoni* at the beginning of a national control program in Uganda in 2003, within a highly endemic region on Lake Victoria, found that although egg output and genetic diversity significantly decreased immediately after drug administration, both recovered within 6 months.^67^ Both these Tanzanian^47^ and Ugandan^67^ studies also observed low genetic differentiation between children from different schools, indicative of high rates of parasite gene flow at this geographic scale. This is likely to reduce the likelihood of local adaptation, so even if repeated drug treatments select resistant genotypes, these may be diluted and outcompeted by new genotypes migrating in. These findings also suggested that resistance to PZQ may be unlikely to establish or spread in these populations at the beginning of the national control program, given the current selection of targeting school-aged children alone.^69^ Nevertheless, these studies on *S. mansoni* susceptibility to PZQ (at least within Uganda), as recent findings have shown for oxamniquine resistance,^66^ have identified standing variation in drug efficacies and clearance rates on which selection can act. This might indeed be expected to accentuate as drug pressures over time increase. It is possible that evidence of such may be apparent already within Uganda, at least, where those populations under the longest and strongest past treatment pressures have been associated with significant reductions in drug efficacy.^15^

Therefore, even without current molecular markers of PZQ resistance, we can demonstrate that quantifying population structure can help predict the likelihood of genotype spread when local populations are under selection, such as by PZQ MDA. The WHO recommends that efficacy be assessed either when there is a suspicion that PZQ is not performing as expected despite satisfactory coverage and adherence or when the drug has been distributed by MDA for 4 years or more.^25^ In practice, this guidance is seldom adopted and the efficacy of PZQ is not monitored on a systematic basis. The fact that some schistosomes do survive exposure to PZQ as it is currently dosed and that PZQ is being distributed on an unprecedented scale should be a strong stimulus to monitor for the possible development of drug resistance on a more routine basis. Indeed, mathematical predictive models already suggest that the increased chemotherapeutic treatment pressures now being dispensed in SSA might allow both parasites with drug resistance/intermediate susceptibility to successfully invade and coexist with susceptible and resistant parasite strains.^71^

### Implications and applications of genetic studies for *Schistosoma* control or elimination in SSA.

Gene flow, effective population sizes, reproductive and mating systems, host transmission and host specificity, and the standing variation in terms of potential resistance alleles, to name but a few, will all affect the epidemiology of schistosomiasis by influencing the evolutionary potential of these parasites in response to the control pressures imposed on them. Understanding these parameters will help predict the likelihood of success while mitigating against the evolution and establishment of drug resistance and increase the longevity and sustainability of interventions. When designing disease control programs, and monitoring and evaluating their impact, it is essential that we can accurately understand and predict parasite populations. Indeed, knowing the population boundaries is required to characterize the units relevant for infection control and the geographical scale at which control measures must be applied. Because of their complex indirectly transmitted life cycle, size, and location within the (human) definitive host, deciphering the patterns of transmission and mating/reproductive patterns of schistosomes requires indirect methods such as population genetic analyses using tools such as microsatellite markers.

Within those SCORE countries and treatment arms encompassing population genetic analyses, further insights have been revealed that could not have been achieved by classical prevalence and intensity parasitological tools alone. We have also observed a basic challenge to genetic studies based on the cluster randomization design in these operational research studies. For example, with both *S. mansoni* on mainland Tanzania and *S. haematobium* in Niger, where we have seen extremely high levels of gene flow, the migration of parasites between treatment arms and villages, and between treated and untreated people, occurs and mitigates against definitive demarcation of treatment pressure impact. By contrast, the potential limited gene flow of *S. haematobium* on the Zanzibar islands may result in contrasting population genetic structures between even geographically close areas, overwhelming any potential treatment arm effects imposed.

Furthermore, there is no general consensus on how to define biological hotspots, based on parasite genetics/responsiveness, as distinct from operational hotspots, even if a broad definition that accounts for any failure to decrease prevalence and/or intensity over time is favored.^72^ However, population genetic analyses on these SCORE datasets to date indicate that, with regard to Tanzanian *S. mansoni* at least, persistence may relate to localized high-transmission water contact sites, whereas in Zanzibar, it may relate more to inherent differences in the fecundity and, hence, egg output of the infecting *S. haematobium* across small geographical scales, potentially independent of the treatment arm. In regard to SCORE studies from Niger, the major factor relating to ongoing transmission sites appeared to be the geographical variation in the occurrence of introgressed hybridized schistosomes between *S. haematobium* with *S. bovis.* Although such hybridization events in Niger may well be independent of the SCORE treatment arms, they do highlight the issue of animal reservoirs of schistosome infection in SSA, and the need for a One Health approach to achieve WHO control and elimination targets.^9,73^

It should be noted that all the SCORE parasite samples are archived within the Schistosomiasis collection at Natural History Museum (SCAN) repository.^74^ This, coupled with the increased availability of genome sequences and the recent advances and affordability of DNA sequencing technologies, will greatly facilitate comparative studies of species/populations from contrasting situations (such as between those in PHS from responder sites). This will further facilitate the identification of loci of particular interest, and population genetic and genomic studies of schistosomes may become even more helpful in determining the impact of differential treatment regimens on schistosomes. In turn, such findings will shed light on what strategies and counterstrategies schistosomes may possess to help maintain and maximize their ongoing transmission. Population genetic and genomic analyses of these complex and resourceful parasites under intervention pressure should help further elucidate fundamental principles of ecological and evolutionary theory from that of host specialization, reproductive mode, transmission dynamics, speciation, and even perhaps the evolution of parasitism itself, as well as providing much needed information for control and elimination efforts.
